# Supplementary material for: Emergent structural correlations in dense liquids
Source: PNAS Nexus. 2023 Jun 19;2(6):pgad184. doi: 10.1093/pnasnexus/pgad184 (PMC10279420; doi:10.1093/pnasnexus/pgad184)
Supplement: pgad184_Supplementary_Data [file pgad184_supplementary_data.pdf]

Supporting Information for

Emergent Structural Correlations in Dense Liquids

Ilian Pihlajamaa, Corentin C. L. Laudicina, Chengjie Luo and Liesbeth M.C. Janssen

Liesbeth M.C. Janssen
E-mail: l.m.c.janssen@tue.nl

This PDF file includes:

Supporting text
Figs. S1 to S3
SI References

Supporting Information Text

Density Functional Theory & Standard Definitions

We closely follow (1). We consider a classical fluid of N particles governed by Hamiltonian $\mathcal{H}$ at inverse temperature β

$$\mathcal{H} = \mathcal{T} + V + \Phi \tag{S1}$$

where $\mathcal{T}$ contains the kinetic contributions, V the total potential energy and Φ some spatially varying field which couples to the system's density and can be written from a potential φ as

$$\Phi = \int d\mathbf{r}\rho(\mathbf{r})\varphi(\mathbf{r}) \tag{S2}$$

where $\rho(\mathbf{r}) = \sum_{j=1}^{N} \delta(\mathbf{r} - \mathbf{r}_j)$. We define the local activity by

$$z(\mathbf{r}) = \Lambda^{-3} \exp[\beta\psi(\mathbf{r})] \tag{S3}$$

where Λ is the thermal de Broglie wavelength and $\psi(\mathbf{r}) = \mu - \varphi(\mathbf{r})$ is a spatially dependent chemical potential. Denoting $\Xi(\mu, V, T)$ the grand partition function (GPF):

$$\Xi(\mu, V, T) \equiv \sum_{N=0}^{\infty} \frac{1}{N!h^{3N}} \int d^3\mathbf{p}^N d^3\mathbf{r}^N \exp\beta(\psi(\mathbf{r}^N) - \mathcal{H}(\mathbf{r}^N, \mathbf{p}^N)). \tag{S4}$$

We can then obtain the grand potential Ω by calculating $\Omega = -\beta^{-1}\ln(\Xi)$.

The n-particle density of the system is then simply given by the n-th functional derivative of GPF with respect to the activity $z(\mathbf{r}_i) \equiv z(i)$ in condensed notation $i \equiv \mathbf{r}_i$. We find

$$\rho^{(n)}(1, ..., n) = \frac{z(1)...z(n)}{\Xi} \frac{\delta^n \Xi}{\delta z(1)...\delta z(n)}. \tag{S5}$$

We define a correlation function $H^{(n)}$ of density fluctuations :

$$\begin{aligned} H^{(n)}(1, ..., n) =& \langle[\rho(1) - \rho^{(1)}(1)] \times ... \times [\rho(n) - \rho^{(1)}(n)]\rangle \\ =& \frac{\delta^n \ln(\Xi)}{\delta \ln(z(1))...\delta \ln(z(n))}. \end{aligned} \tag{S6}$$

The (functional) inverses $K^{(n)}$ to $H^{(n)}$ are also of interest to us. Indeed the integral representation of the inverse relationship for $n = 2$ provides direct access to generalised Ornstein-Zernike relations from which convolution approximations to many-body structure factors can be derived, as will be shown below. In order to appropriately define $K^{(n)}$, we consider the Legendre transform of the grand potential and write the free energy $\mathcal{F}$ as

$$\mathcal{F}[\rho^{(1)}] = \Omega[\psi] + \int d\mathbf{r}\rho^{(1)}(\mathbf{r})\psi(\mathbf{r}), \tag{S7}$$

which turns out to be the generating functional for the set of (inverse) n-particle functions

$$K^{(n)}(1, ..., n) = -\frac{\delta^n \beta\mathcal{F}}{\delta\rho^{(1)}(1)...\delta\rho^{(1)}(n)}. \tag{S8}$$

We can decompose the free-energy function $\mathcal{F} = \mathcal{F}_{\text{id}} + \mathcal{F}_{\text{exc}}$ into an ideal and an excess contribution. The ideal contribution reads

$$\beta\mathcal{F}_{\text{id}}[\rho^{(1)}] = \int d\mathbf{r}\rho^{(1)}(\mathbf{r}) \left[\ln(\rho^{(1)}(\mathbf{r})\Lambda^3) - 1\right] \tag{S9}$$

and consists of purely entropic contributions to the free energy, while the excess free energy includes contributions from the interactions (both inter-particle potentials and possibly external potentials). We find for the ideal contribution that (for $n \geq 2$):

$$\begin{aligned} -\frac{\delta^n \beta\mathcal{F}_{\text{id}}}{\delta\rho^{(1)}(1)...\delta\rho^{(1)}(n)} &= -\frac{\delta^n}{\delta\rho^{(1)}(1)...\delta\rho^{(1)}(n)} \int d\mathbf{r}\rho^{(1)}(\mathbf{r}) \left[\ln(\rho^{(1)}(\mathbf{r})\Lambda^3) - 1\right] \\ &= \frac{(-1)^n(n-2)!}{[\rho^{(1)}(1)]^{n-1}} \delta(1, 2) \times ... \times \delta(1, n), \end{aligned} \tag{S10}$$

where $\delta(a, b) \equiv \delta(\mathbf{r}_a - \mathbf{r}_b)$ in the condensed notation previously defined. The excess part of the functions $K^{(n)}$ is used to define the n-particle direct correlation functions $c^{(n)}$:

$$c^{(n)}(1,...,n) = -\frac{\delta^n \beta \mathcal{F}_{\mathrm{exc}}}{\delta\rho^{(1)}(1)...\delta\rho^{(1)}(n)}, \tag{S11}$$

which naturally satisfy the property

$$c^{(n)}(1,...,n) = \frac{\delta c^{(n-1)}(1,...,n-1)}{\delta\rho^{(1)}(n)}. \tag{S12}$$

By construction then, the direct correlation functions encapsulate the effect of interactions on many-particle distribution functions. We may write in full that (for $n \geq 2$)

$$K^{(n)}(1,...,n) = \frac{(-1)^n(n-2)!}{[\rho^{(1)}(1)]^{n-1}}\delta(1,2) \times ... \times \delta(1,n) - c^{(n)}(1,...,n). \tag{S13}$$

For the initial case $n = 2$, we can write down the following functional identity

$$\int d3 K^{(2)}(1,3)H^{(2)}(3,2) = \delta(1,2). \tag{S14}$$

By taking repeated functional derivatives with respect to the one-body density field $\rho^{(1)}$ of Eq. Eq. (S14) above, we iteratively obtain generalised Ornstein-Zernike integral equations by substituting in the definitions for $H^{(n)}$ and $K^{(n)}$. As a last remark we note that we often work in reciprocal Fourier space, it is thus important to realise that: $H^{(n)}(1,...,n) = \rho_0 S^{(n)}(1,...,n)$ where $S^{(n)}(1,...,n) = S^{(n)}(\mathbf{k}_1,...,\mathbf{k}_n) = N^{-1}\langle\hat{\rho}_{\mathbf{k}_1}...\hat{\rho}_{\mathbf{k}_n}\rangle$ (1) is the n-body static structure factor. Additionally, we find that

$$K^{(n)}(\mathbf{k}_1,...,\mathbf{k}_n) = \frac{(-1)^n(n-2)!}{\rho_0^{n-1}} - c^{(n)}(\mathbf{k}_1,...,\mathbf{k}_n). \tag{S15}$$

By restricting to a homogeneous fluid we require that $\sum_{i=1}^n \mathbf{k}_j = 0$, i.e. momentum is a conserved quantity. The relation above in reciprocal space reads after enforcing translational and rotational invariance:

$$\begin{aligned}
\int d1d2 e^{-i\mathbf{k}_1\cdot 1 - i\mathbf{k}_2\cdot 2}\delta(1-2) &= \int d1d2 e^{-i\mathbf{k}_1\cdot 1 - i\mathbf{k}_2\cdot 2}\int d3 K^{(2)}(1-3)H^{(2)}(3-2) \\
\delta(\mathbf{k}_1 - \mathbf{k}_2) &= \int d13d32 \int d3 e^{-i\mathbf{k}_1\cdot 13}K^{(2)}(13)e^{i\mathbf{k}_2\cdot 32}H^{(2)}(32)e^{i(\mathbf{k}_1-\mathbf{k}_2)\cdot 3} \\
\delta(\mathbf{k}_1 - \mathbf{k}_2) &= K^{(2)}(|\mathbf{k}_1|)H^{(2)}(|\mathbf{k}_2|)\delta(\mathbf{k}_1 - \mathbf{k}_2),
\end{aligned} \tag{S16}$$

so that $1 = K^{(2)}(k)H^{(2)}(k)$ and thus $K^{(2)}(k) = \rho_0^{-1}(S^{(2)})^{-1}(k)$.

A. Generalised Ornstein Zernike Equations

A. Single Component Fluids. From Eq. Eq. (S14) we systematically consider functional derivatives with respect to the one-body density to derive generalised Ornstein-Zernike integral equations. By enforcing translational and rotational invariance we obtain algebraic relations in reciprocal space. By substituting the definitions of $K^{(n)}$ and $H^{(n)}$, we obtain formally exact equations for the direct correlation function $c^{(n)}$ from which approximate expressions for the many-body static structure factors $S^{(n)}$ can be derived from (simple) algebraic inversion.

A.1. Three-body correlation functions : $c^{(3)}$ and $S^{(3)}$. We consider the functional derivative of Eq. Eq. (S14) w.r.t. $\rho^{(1)}(4)$. This gives:

$$\begin{aligned}
0 = \frac{\delta}{\delta\rho^{(1)}(4)}\int d3 K^{(2)}(1,3)H^{(2)}(3,2) &= \int d3 K^{(3)}(1,3,4)H^{(2)}(3,2) + \int d3 K^{(2)}(1,3)\frac{\delta H^{(2)}(3,2)}{\delta\rho^{(1)}(4)} \\
&= \int d3 K^{(3)}(1,3,4)H^{(2)}(3,2) + \int d3 K^{(2)}(1,3)\int d5 \frac{\delta H^{(2)}(3,2)}{\delta \ln(z(5))}\frac{\delta \ln(z(5))}{\delta\rho^{(1)}(4)} \\
&= \int d3 K^{(3)}(1,3,4)H^{(2)}(3,2) + \int d3d5 K^{(2)}(1,3)H^{(3)}(3,2,5)K^{(2)}(5,4).
\end{aligned}$$

So that we find

$$\int d3 K^{(3)}(1,3,4)H^{(2)}(3,2) + \int d3d5 K^{(2)}(1,3)H^{(3)}(3,2,5)K^{(2)}(5,4) = 0, \tag{S17}$$

which constitutes the next equation in the hierarchy of generalised OZ relations. Enforcing translational and rotational invariance, we find (now using complete notation for clarity)

$$\int d\mathbf{r}_3 K^{(3)}(\mathbf{r}_3-\mathbf{r}_1,\mathbf{r}_3-\mathbf{r}_4)H^{(2)}(\mathbf{r}_3-\mathbf{r}_2)+\int d\mathbf{r}_3 d\mathbf{r}_5 K^{(2)}(\mathbf{r}_3-\mathbf{r}_1)H^{(3)}(\mathbf{r}_3-\mathbf{r}_2,\mathbf{r}_3-\mathbf{r}_5)K^{(2)}(\mathbf{r}_4-\mathbf{r}_5)=0. \quad \text{[S18]}$$

Fourier transformation gives for the first term of Eq. Eq. (S18)

$$\begin{aligned}&\int d\mathbf{r}_1 d\mathbf{r}_2 d\mathbf{r}_3 \mathbf{r}_4 e^{-i\mathbf{k}_1\cdot(\mathbf{r}_1-\mathbf{r}_3)}e^{-i\mathbf{k}_2\cdot(\mathbf{r}_2-\mathbf{r}_3)}e^{-i\mathbf{k}_4\cdot(\mathbf{r}_4-\mathbf{r}_3)}K^{(3)}(\mathbf{r}_3-\mathbf{r}_1,\mathbf{r}_3-\mathbf{r}_4)H^{(2)}(\mathbf{r}_3-\mathbf{r}_2)e^{-i(\mathbf{k}_1+\mathbf{k}_2+\mathbf{k}_4)\cdot\mathbf{r}_3}\\&=K^{(3)}(-\mathbf{k}_1,-\mathbf{k}_4)H^{(2)}(-\mathbf{k}_2)\delta(\mathbf{k}_1+\mathbf{k}_2+\mathbf{k}_4),\end{aligned}$$

while the second term of Eq. Eq. (S18) gives

$$\begin{aligned}&\int d\mathbf{r}_1...d\mathbf{r}_5 e^{-i\mathbf{k}_1\cdot(\mathbf{r}_1-\mathbf{r}_3)}K^{(2)}(\mathbf{r}_3-\mathbf{r}_1)e^{-i\mathbf{k}_2\cdot(\mathbf{r}_2-\mathbf{r}_3)}e^{-i\mathbf{k}_4\cdot(\mathbf{r}_5-\mathbf{r}_3)}H^{(3)}(\mathbf{r}_3-\mathbf{r}_2,\mathbf{r}_3-\mathbf{r}_5)e^{-i\mathbf{k}_4\cdot(\mathbf{r}_4-\mathbf{r}_5)}K^{(2)}(\mathbf{r}_4-\mathbf{r}_5)\\&\times e^{-i(\mathbf{k}_1+\mathbf{k}_2+\mathbf{k}_4)\cdot\mathbf{r}_3}\\&=K^{(2)}(-\mathbf{k}_1)H^{(3)}(-\mathbf{k}_2,-\mathbf{k}_4)K^{(2)}(\mathbf{k}_4)\delta(\mathbf{k}_1+\mathbf{k}_2+\mathbf{k}_4).\end{aligned}$$

We note that technically we can write $H^{(3)}(-\mathbf{k}_2,-\mathbf{k}_4)=H^{(3)}(-\mathbf{k}_2,-\mathbf{k}_4,-\mathbf{k}_1)=H^{(3)}(-\mathbf{k}_1,-\mathbf{k}_4)$ since one of the three variables is redundant by momentum conservation. This is a useful ‘trick’ that allows for simplifications and symmetrisations of the generalised OZ equations in reciprocal space. This also allows us to write $K^{(3)}(-\mathbf{k}_1,-\mathbf{k}_4)=K^{(3)}(-\mathbf{k}_1,-\mathbf{k}_2)$ in the first term. Finally after recombining the expression and letting $\mathbf{k}_{1,2}\to-\mathbf{k}_{1,2}$, we find

$$K^{(3)}(\mathbf{k}_1,\mathbf{k}_2)H^{(2)}(\mathbf{k}_2)+K^{(2)}(\mathbf{k}_1)H^{(3)}(\mathbf{k}_1,\mathbf{k}_2)K^{(2)}(\mathbf{k}_1+\mathbf{k}_2)=0. \quad \text{[S19]}$$

Substituting the expressions for $K^{(n)}$ in terms of $c^{(n)}$ and that of $H^{(n)}$ in terms of $S^{(n)}$, we find

$$S^{(3)}(\mathbf{k}_1,\mathbf{k}_2)=S^{(2)}(|\mathbf{k}_1|)S^{(2)}(|\mathbf{k}_2|)S^{(2)}(|\mathbf{k}_1+\mathbf{k}_2|)\left[1+\rho_0^2c^{(3)}(\mathbf{k}_1,\mathbf{k}_2),\right] \quad \text{[S20]}$$

which is the defining relation for $c^{(3)}$. We note that by neglecting the direct correlation function : $c^{(3)}=0$, we recover the standard convolution approximation

$$S^{(3)}(\mathbf{k}_1,\mathbf{k}_2)\approx S^{(2)}(|\mathbf{k}_1|)S^{(2)}(|\mathbf{k}_2|)S^{(2)}(|\mathbf{k}_1+\mathbf{k}_2|) \quad \text{[S21]}$$

used in many theories of the liquid state.

A.2. Four-body correlation functions : $c^{(4)}$ and $S^{(4)}$. We now extend the previous calculation to the next order and derive an exact equation for the 4-point direct correlation function. Similarly, an approximate convolution approximation for the 4-body static structure factor can be obtained. We note that the expression for $S^{(4)}$ has previously been derived and used in the derivation of the inhomogeneous mode-coupling theory of the glass transition (2). We start by taking the functional derivative of Eq. Eq. (S18) with respect to $\rho^{(1)}(5)$, which gives, after using the same functional chain-rule decomposition $\delta H^{(n)}/\delta\rho=\delta H^{(n)}/\delta\ln(z)*\delta\ln(z)/\delta\rho^{(1)}=H^{(n+1)}*K^{(2)}$:

$$\begin{aligned}0=&\int d5K^{(4)}(1,5,2,3)H^{(2)}(5,4)+\int d5d6K^{(3)}(1,5,2)H^{(3)}(5,4,6)K^{(2)}(6,3)\\&+\int d5d6K^{(3)}(1,5,3)H^{(3)}(5,4,6)K^{(2)}(6,2)\\&+\int d5d6d7K^{(2)}(1,5)H^{(4)}(5,4,6,7)K^{(2)}(6,3)K^{(2)}(7,2)\\&+\int d5d6K^{(2)}(1,5)H^{(3)}(5,4,6)K^{(3)}(6,2,3),\end{aligned} \quad \text{[S22]}$$

which in Fourier reciprocal space will read

$$\begin{aligned}0=&K^{(4)}(\mathbf{k}_1,\mathbf{k}_2,\mathbf{k}_3)H^{(2)}(\mathbf{k}_1+\mathbf{k}_2+\mathbf{k}_3)+K^{(3)}(\mathbf{k}_1,\mathbf{k}_2)H^{(3)}(\mathbf{k}_1+\mathbf{k}_2,\mathbf{k}_3)K^{(2)}(\mathbf{k}_3)\\&+K^{(3)}(\mathbf{k}_1,\mathbf{k}_3)H^{(3)}(\mathbf{k}_1+\mathbf{k}_3,\mathbf{k}_2)K^{(2)}(\mathbf{k}_2)+K^{(2)}(\mathbf{k}_1)H^{(4)}(\mathbf{k}_1,\mathbf{k}_2,\mathbf{k}_3)K^{(2)}(\mathbf{k}_3)K^{(2)}(\mathbf{k}_2)\\&+K^{(2)}(\mathbf{k}_1)H^{(3)}(\mathbf{k}_1,\mathbf{k}_2+\mathbf{k}_3)K^{(3)}(\mathbf{k}_2,\mathbf{k}_3).\end{aligned} \quad \text{[S23]}$$

Now substituting for the definitions of $K^{(n)}$ and $H^{(n)}$ and rearranging for $S^{(4)}$, we obtain

$$
\begin{aligned}
S^{(4)}(\mathbf{k}_1,\mathbf{k}_2,\mathbf{k}_3) = {} & S^{(2)}(|\mathbf{k}_1|)S^{(2)}(|\mathbf{k}_2|)S^{(2)}(|\mathbf{k}_3|) \\
& \times \Bigg\{ \left(1+\rho_0^2 c^{(3)}(\mathbf{k}_1,\mathbf{k}_2)\right) \frac{S^{(3)}(\mathbf{k}_1+\mathbf{k}_2,\mathbf{k}_3)}{S^{(2)}(|\mathbf{k}_3|)} \\
& \quad + \left(1+\rho_0^2 c^{(3)}(\mathbf{k}_1,\mathbf{k}_3)\right) \frac{S^{(3)}(\mathbf{k}_1+\mathbf{k}_3,\mathbf{k}_2)}{S^{(2)}(|\mathbf{k}_2|)} \\
& \quad + \left(1+\rho_0^2 c^{(3)}(\mathbf{k}_2,\mathbf{k}_3)\right) \frac{S^{(3)}(\mathbf{k}_2+\mathbf{k}_3,\mathbf{k}_1)}{S^{(2)}(|\mathbf{k}_1|)} \\
& \quad - \left(2-\rho_0^3 c^{(4)}(\mathbf{k}_1,\mathbf{k}_2,\mathbf{k}_3)\right) S^{(2)}(|\mathbf{k}_1+\mathbf{k}_2+\mathbf{k}_3|) \Bigg\}.
\end{aligned}
\tag{S24}
$$

Again, by neglecting all $c^{(n)}$, i.e., $c^{(n)}=0$ for $n=3,4$, and using the convolution approximation for the $S^{(3)}$ [Eq. Eq. (S21)], we obtain a convolution approximation for the 4-body static structure factor:

$$
S^{(4)}(\mathbf{k}_1,\mathbf{k}_2,\mathbf{k}_3) \approx S^{(2)}(|\mathbf{k}_1|)S^{(2)}(|\mathbf{k}_2|)S^{(2)}(|\mathbf{k}_3|)S^{(2)}(|\mathbf{k}_1+\mathbf{k}_2+\mathbf{k}_3|)\left[S^{(2)}(|\mathbf{k}_1+\mathbf{k}_2|)+S^{(2)}(|\mathbf{k}_1+\mathbf{k}_3|)+S^{(2)}(|\mathbf{k}_2+\mathbf{k}_3|)-2\right],
\tag{S25}
$$

which is clearly symmetric under exchange of wave-vectors, as required.

A.3. Five-body correlation functions : $c^{(5)}$ and $S^{(5)}$. The procedure is the same for order 5. We only show the final result for the convolution approximation (that is after neglecting all $c^{(n)}$) :

$$
\begin{aligned}
S^{(5)}(\mathbf{k}_1,\mathbf{k}_2,\mathbf{k}_3,\mathbf{k}_4,\mathbf{k}_5) \approx {} & S^{(2)}(\mathbf{k}_1)S^{(2)}(\mathbf{k}_2)S^{(2)}(\mathbf{k}_3)S^{(2)}(\mathbf{k}_5)\Bigg\{6S^{(2)}(\mathbf{k}_4) - 2\Bigg(\frac{S^{(3)}(\mathbf{k}_4,\mathbf{k}_1+\mathbf{k}_2+\mathbf{k}_3)}{S^{(2)}(\mathbf{k}_5)} + \frac{S^{(3)}(\mathbf{k}_1,\mathbf{k}_4)}{S^{(2)}(\mathbf{k}_1)} \\
& + \frac{S^{(3)}(\mathbf{k}_2,\mathbf{k}_4)}{S^{(2)}(\mathbf{k}_2)} + \frac{S^{(3)}(\mathbf{k}_3,\mathbf{k}_4)}{S^{(2)}(\mathbf{k}_3)}\Bigg) - S^{(3)}(\mathbf{k}_1+\mathbf{k}_2,\mathbf{k}_4) - S^{(3)}(\mathbf{k}_1+\mathbf{k}_3,\mathbf{k}_4) - S^{(3)}(\mathbf{k}_2+\mathbf{k}_3,\mathbf{k}_4) \\
& + \frac{1}{S^{(2)}(\mathbf{k}_5)}\Bigg(\frac{S^{(4)}(\mathbf{k}_1+\mathbf{k}_2,\mathbf{k}_3,\mathbf{k}_4)}{S^{(2)}(\mathbf{k}_3)} + \frac{S^{(4)}(\mathbf{k}_1+\mathbf{k}_3,\mathbf{k}_2,\mathbf{k}_4)}{S^{(2)}(\mathbf{k}_2)} + \frac{S^{(4)}(\mathbf{k}_2+\mathbf{k}_3,\mathbf{k}_1,\mathbf{k}_4)}{S^{(2)}(\mathbf{k}_1)}\Bigg) \\
& + \frac{S^{(4)}(\mathbf{k}_2,\mathbf{k}_3,\mathbf{k}_4)}{S^{(2)}(\mathbf{k}_2)S^{(2)}(\mathbf{k}_3)} + \frac{S^{(4)}(\mathbf{k}_1,\mathbf{k}_2,\mathbf{k}_4)}{S^{(2)}(\mathbf{k}_1)S^{(2)}(\mathbf{k}_2)} + \frac{S^{(4)}(\mathbf{k}_1,\mathbf{k}_3,\mathbf{k}_4)}{S^{(2)}(\mathbf{k}_1)S^{(2)}(\mathbf{k}_3)}\Bigg\}
\end{aligned}
\tag{S26}
$$

which can be further simplified to

$$
\begin{aligned}
S^{(5)}(\mathbf{k}_1,\mathbf{k}_2,\mathbf{k}_3,\mathbf{k}_4,\mathbf{k}_5) \approx {} & S^{(2)}(\mathbf{k}_1)S^{(2)}(\mathbf{k}_2)S^{(2)}(\mathbf{k}_3)S^{(2)}(\mathbf{k}_4)S^{(2)}(\mathbf{k}_1+\mathbf{k}_2+\mathbf{k}_3+\mathbf{k}_4) \\
& \Bigg(S^{(2)}(\mathbf{k}_2+\mathbf{k}_3)S^{(2)}(\mathbf{k}_1+\mathbf{k}_2+\mathbf{k}_3) + S^{(2)}(\mathbf{k}_2+\mathbf{k}_3)S^{(2)}(\mathbf{k}_1+\mathbf{k}_4) \\
& + S^{(2)}(\mathbf{k}_1+\mathbf{k}_4)S^{(2)}(\mathbf{k}_1+\mathbf{k}_2+\mathbf{k}_4) + S^{(2)}(\mathbf{k}_2+\mathbf{k}_4)S^{(2)}(\mathbf{k}_1+\mathbf{k}_2+\mathbf{k}_4) \\
& + S^{(2)}(\mathbf{k}_1+\mathbf{k}_2)\{-2+S^{(2)}(\mathbf{k}_1+\mathbf{k}_2+\mathbf{k}_3)+S^{(2)}(\mathbf{k}_1+\mathbf{k}_2+\mathbf{k}_4)+S^{(2)}(\mathbf{k}_3+\mathbf{k}_4)\} \\
& + S^{(2)}(\mathbf{k}_1+\mathbf{k}_4)S^{(2)}(\mathbf{k}_1+\mathbf{k}_3+\mathbf{k}_4) + S^{(2)}(\mathbf{k}_3+\mathbf{k}_4)S^{(2)}(\mathbf{k}_1+\mathbf{k}_3+\mathbf{k}_4) \\
& + S^{(2)}(\mathbf{k}_1+\mathbf{k}_3)\{-2+S^{(2)}(\mathbf{k}_1+\mathbf{k}_2+\mathbf{k}_3)+S^{(2)}(\mathbf{k}_2+\mathbf{k}_4)+S^{(2)}(\mathbf{k}_1+\mathbf{k}_3+\mathbf{k}_4)\} \\
& + S^{(2)}(\mathbf{k}_2+\mathbf{k}_3+\mathbf{k}_4)\left[S^{(2)}(\mathbf{k}_2+\mathbf{k}_3)+S^{(2)}(\mathbf{k}_2+\mathbf{k}_4)+S^{(2)}(\mathbf{k}_3+\mathbf{k}_4)\right] \\
& - 2\Bigg[-3+S^{(2)}(\mathbf{k}_2+\mathbf{k}_3)+S^{(2)}(\mathbf{k}_1+\mathbf{k}_2+\mathbf{k}_3)+S^{(2)}(\mathbf{k}_1+\mathbf{k}_4) \\
& + S^{(2)}(\mathbf{k}_2+\mathbf{k}_4)+S^{(2)}(\mathbf{k}_1+\mathbf{k}_2+\mathbf{k}_4)+S^{(2)}(\mathbf{k}_3+\mathbf{k}_4)+S^{(2)}(\mathbf{k}_1+\mathbf{k}_3+\mathbf{k}_4)+S^{(2)}(\mathbf{k}_2+\mathbf{k}_3+\mathbf{k}_4)\Bigg]\Bigg)
\end{aligned}
\tag{S27}
$$

where for clarity we have omitted absolute values in the function arguments, but $S^{(2)}(\mathbf{k}) \equiv S^{(2)}(|\mathbf{k}|)$ should be clear from context. We remark that the expression is symmetric under exchange of wave-vector arguments, as should be the case.

A.4. Six-body correlation functions : $c^{(6)}$ ***and*** $S^{(6)}$***.*** The procedure is the same for order 6. We only show the final result for the convolution approximation (that is after neglecting all $c^{(n)}$):

$$
\begin{aligned}
&S^{(6)}(\mathbf{k}_1,\mathbf{k}_2,\mathbf{k}_3,\mathbf{k}_4,\mathbf{k}_5)\\
\approx &S^{(2)}(\mathbf{k}_2)S^{(2)}(\mathbf{k}_3)S^{(2)}(\mathbf{k}_4)S^{(2)}(\mathbf{k}_5)S^{(2)}(\mathbf{k}_6)\Bigg\{-24S^{(2)}(\mathbf{k}_1)+6\Bigg(\frac{S^{(3)}(\mathbf{k}_1,\mathbf{k}_5)}{S^{(2)}(\mathbf{k}_5)}+\frac{S^{(3)}(\mathbf{k}_1,\mathbf{k}_4)}{S^{(2)}(\mathbf{k}_4)}+\frac{S^{(3)}(\mathbf{k}_1,\mathbf{k}_3)}{S^{(2)}(\mathbf{k}_3)}\\
&+\frac{S^{(3)}(\mathbf{k}_1,\mathbf{k}_2)}{S^{(2)}(\mathbf{k}_2)}+\frac{S^{(3)}(\mathbf{k}_1,\mathbf{k}_2+\mathbf{k}_3+\mathbf{k}_4+\mathbf{k}_5)}{S^{(2)}(\mathbf{k}_6)}\Bigg)+2\Bigg(S^{(3)}(\mathbf{k}_1,\mathbf{k}_4+\mathbf{k}_5)+S^{(3)}(\mathbf{k}_1,\mathbf{k}_3+\mathbf{k}_5)\\
&+S^{(3)}(\mathbf{k}_1,\mathbf{k}_3+\mathbf{k}_4)+S^{(3)}(\mathbf{k}_1,\mathbf{k}_3+\mathbf{k}_4+\mathbf{k}_5)+S^{(3)}(\mathbf{k}_1,\mathbf{k}_2+\mathbf{k}_5)+S^{(3)}(\mathbf{k}_1,\mathbf{k}_2+\mathbf{k}_4)\\
&+S^{(3)}(\mathbf{k}_1,\mathbf{k}_2+\mathbf{k}_4+\mathbf{k}_5)+S^{(3)}(\mathbf{k}_1,\mathbf{k}_2+\mathbf{k}_3)+S^{(3)}(\mathbf{k}_1,\mathbf{k}_2+\mathbf{k}_3+\mathbf{k}_5)+S^{(3)}(\mathbf{k}_1,\mathbf{k}_2+\mathbf{k}_3+\mathbf{k}_4)\Bigg)\\
&-2\Bigg(\frac{S^{(4)}(\mathbf{k}_1,\mathbf{k}_4,\mathbf{k}_5)}{S^{(2)}(\mathbf{k}_4)S^{(2)}(\mathbf{k}_5)}+\frac{S^{(4)}(\mathbf{k}_1,\mathbf{k}_3,\mathbf{k}_5)}{S^{(2)}(\mathbf{k}_3)S^{(2)}(\mathbf{k}_5)}+\frac{S^{(4)}(\mathbf{k}_1,\mathbf{k}_3,\mathbf{k}_4)}{S^{(2)}(\mathbf{k}_3)S^{(2)}(\mathbf{k}_4)}+\frac{S^{(4)}(\mathbf{k}_1,\mathbf{k}_2,\mathbf{k}_5)}{S^{(2)}(\mathbf{k}_2)S^{(2)}(\mathbf{k}_5)}+\frac{S^{(4)}(\mathbf{k}_1,\mathbf{k}_2,\mathbf{k}_4)}{S^{(2)}(\mathbf{k}_4)S^{(2)}(\mathbf{k}_2)}\\
&+\frac{S^{(4)}(\mathbf{k}_1,\mathbf{k}_2,\mathbf{k}_3)}{S^{(2)}(\mathbf{k}_2)S^{(2)}(\mathbf{k}_3)}+\frac{S^{(4)}(\mathbf{k}_1,\mathbf{k}_2,\mathbf{k}_3+\mathbf{k}_4+\mathbf{k}_5)}{S^{(2)}(\mathbf{k}_2)S^{(2)}(\mathbf{k}_6)}+\frac{S^{(4)}(\mathbf{k}_1,\mathbf{k}_3,\mathbf{k}_2+\mathbf{k}_4+\mathbf{k}_5)}{S^{(2)}(\mathbf{k}_3)S^{(2)}(\mathbf{k}_6)}+\frac{S^{(4)}(\mathbf{k}_1,\mathbf{k}_4,\mathbf{k}_2+\mathbf{k}_3+\mathbf{k}_5)}{S^{(2)}(\mathbf{k}_4)S^{(2)}(\mathbf{k}_6)}\\
&+\frac{S^{(4)}(\mathbf{k}_1,\mathbf{k}_5,\mathbf{k}_2+\mathbf{k}_3+\mathbf{k}_4)}{S^{(2)}(\mathbf{k}_5)S^{(2)}(\mathbf{k}_6)}\Bigg)-\Bigg(\frac{S^{(4)}(\mathbf{k}_1,\mathbf{k}_3,\mathbf{k}_4+\mathbf{k}_5)}{S^{(2)}(\mathbf{k}_3)}+\frac{S^{(4)}(\mathbf{k}_1,\mathbf{k}_4,\mathbf{k}_3+\mathbf{k}_5)}{S^{(2)}(\mathbf{k}_4)}\\
&+\frac{S^{(4)}(\mathbf{k}_1,\mathbf{k}_5,\mathbf{k}_3+\mathbf{k}_4)}{S^{(2)}(\mathbf{k}_5)}+\frac{S^{(4)}(\mathbf{k}_1,\mathbf{k}_2,\mathbf{k}_4+\mathbf{k}_5)}{S^{(2)}(\mathbf{k}_2)}+\frac{S^{(4)}(\mathbf{k}_1,\mathbf{k}_4,\mathbf{k}_2+\mathbf{k}_5)}{S^{(2)}(\mathbf{k}_4)}+\frac{S^{(4)}(\mathbf{k}_1,\mathbf{k}_5,\mathbf{k}_2+\mathbf{k}_4)}{S^{(2)}(\mathbf{k}_5)}\\
&+\frac{S^{(4)}(\mathbf{k}_1,\mathbf{k}_2,\mathbf{k}_3+\mathbf{k}_5)}{S^{(2)}(\mathbf{k}_2)}+\frac{S^{(4)}(\mathbf{k}_1,\mathbf{k}_3,\mathbf{k}_2+\mathbf{k}_5)}{S^{(2)}(\mathbf{k}_3)}+\frac{S^{(4)}(\mathbf{k}_1,\mathbf{k}_2,\mathbf{k}_3+\mathbf{k}_4)}{S^{(2)}(\mathbf{k}_2)}+\frac{S^{(4)}(\mathbf{k}_1,\mathbf{k}_3+\mathbf{k}_4,\mathbf{k}_2+\mathbf{k}_5)}{S^{(2)}(\mathbf{k}_6)}\\
&+\frac{S^{(4)}(\mathbf{k}_1,\mathbf{k}_3,\mathbf{k}_2+\mathbf{k}_4)}{S^{(2)}(\mathbf{k}_3)}+\frac{S^{(4)}(\mathbf{k}_1,\mathbf{k}_3+\mathbf{k}_5,\mathbf{k}_2+\mathbf{k}_4)}{S^{(2)}(\mathbf{k}_6)}+\frac{S^{(4)}(\mathbf{k}_1,\mathbf{k}_2+\mathbf{k}_3,\mathbf{k}_5)}{S^{(2)}(\mathbf{k}_5)}+\frac{S^{(4)}(\mathbf{k}_1,\mathbf{k}_4,\mathbf{k}_2+\mathbf{k}_3)}{S^{(2)}(\mathbf{k}_4)}\\
&+\frac{S^{(4)}(\mathbf{k}_1,\mathbf{k}_2+\mathbf{k}_3,\mathbf{k}_4+\mathbf{k}_5)}{S^{(2)}(\mathbf{k}_6)}\Bigg)+\frac{S^{(5)}(\mathbf{k}_1,\mathbf{k}_2,\mathbf{k}_4,\mathbf{k}_5)}{S^{(2)}(\mathbf{k}_2)S^{(2)}(\mathbf{k}_4)S^{(2)}(\mathbf{k}_5)}+\frac{S^{(5)}(\mathbf{k}_1,\mathbf{k}_2,\mathbf{k}_3,\mathbf{k}_5)}{S^{(2)}(\mathbf{k}_2)S^{(2)}(\mathbf{k}_3)S^{(2)}(\mathbf{k}_5)}+\frac{S^{(5)}(\mathbf{k}_1,\mathbf{k}_2,\mathbf{k}_3,\mathbf{k}_4)}{S^{(2)}(\mathbf{k}_2)S^{(2)}(\mathbf{k}_3)S^{(2)}(\mathbf{k}_4)}\\
&+\frac{S^{(5)}(\mathbf{k}_1,\mathbf{k}_2,\mathbf{k}_3,\mathbf{k}_4+\mathbf{k}_5)}{S^{(2)}(\mathbf{k}_2)S^{(2)}(\mathbf{k}_3)S^{(2)}(\mathbf{k}_6)}+\frac{S^{(5)}(\mathbf{k}_1,\mathbf{k}_2,\mathbf{k}_4,\mathbf{k}_3+\mathbf{k}_5)}{S^{(2)}(\mathbf{k}_2)S^{(2)}(\mathbf{k}_4)S^{(2)}(\mathbf{k}_6)}+\frac{S^{(5)}(\mathbf{k}_1,\mathbf{k}_3,\mathbf{k}_4,\mathbf{k}_2+\mathbf{k}_5)}{S^{(2)}(\mathbf{k}_3)S^{(2)}(\mathbf{k}_4)S^{(2)}(\mathbf{k}_6)}\\
&+\frac{S^{(5)}(\mathbf{k}_1,\mathbf{k}_2,\mathbf{k}_5,\mathbf{k}_3+\mathbf{k}_4)}{S^{(2)}(\mathbf{k}_6)S^{(2)}(\mathbf{k}_2)S^{(2)}(\mathbf{k}_5)}+\frac{S^{(5)}(\mathbf{k}_1,\mathbf{k}_2+\mathbf{k}_3,\mathbf{k}_4,\mathbf{k}_5)}{S^{(2)}(\mathbf{k}_4)S^{(2)}(\mathbf{k}_5)S^{(2)}(\mathbf{k}_6)}+\frac{S^{(5)}(\mathbf{k}_1,\mathbf{k}_3,\mathbf{k}_5,\mathbf{k}_2+\mathbf{k}_4)}{S^{(2)}(\mathbf{k}_6)S^{(2)}(\mathbf{k}_3)S^{(2)}(\mathbf{k}_5)}+\frac{S^{(5)}(\mathbf{k}_1,\mathbf{k}_3,\mathbf{k}_4,\mathbf{k}_5)}{S^{(2)}(\mathbf{k}_3)S^{(2)}(\mathbf{k}_4)S^{(2)}(\mathbf{k}_5)}\Bigg\}.
\end{aligned}
\tag{S28}
$$

B. Multicomponent Fluids.

B.1. Extension of Basis Functions. In order to deal with multicomponent systems we extend the previous basis functions $H^{(n)}$ and $K^{(n)}$ appropriately. To do so we need to promote each basis to a 'tensor' of order n. Naturally, this extension reads

$$H^{(n)}_{\alpha_1\ldots\alpha_n}(1,...,n) = \frac{\delta^n \ln(\Xi)}{\delta \ln(z_{\alpha_1}(1))...\delta \ln(z_{\alpha_n}(n))} \tag{S29}$$

while

$$\begin{aligned} K^{(n)}_{\alpha_1\ldots\alpha_n}(1,...,n) &= \frac{\delta^n \beta\mathcal{F}}{\delta\rho^{(1)}_{\alpha_1}(1)...\delta\rho^{(1)}_{\alpha_n}(n)} \\ &= \frac{(-1)^n(n-2)!}{[\rho^{(1)}_{\alpha_1}(1)]^{n-1}}\delta_{\alpha_1\alpha_2}(1,2)...\delta_{\alpha_1\alpha_n}(1,n) - c^{(n)}_{\alpha_1\ldots\alpha_n}(1,...,n) \end{aligned} \tag{S30}$$

where we have generalised the Kronecker symbol : $\delta_{\alpha\beta}(1,2) \equiv \delta_{\alpha\beta}\delta(\mathbf{r}_1 - \mathbf{r}_2)$. As before we will work in Fourier reciprocal space and thus we write (after assuming translational and rotational invariance)

$$H^{(n)}_{\alpha_1\ldots\alpha_n}(\mathbf{k}_1,...,\mathbf{k}_{n-1}) = \rho_0 S^{(n)}_{\alpha_1\ldots\alpha_n}(\mathbf{k}_1,...,\mathbf{k}_{n-1}) \tag{S31}$$

while

$$K^{(n)}_{\alpha_1\ldots\alpha_n}(\mathbf{k}_1,...,\mathbf{k}_{n-1}) = \frac{1}{\rho_0^{n-1}}\left[\frac{(-1)^n(n-2)!}{x^{n-1}_{\alpha_1}}\delta_{\alpha_1\alpha_2}...\delta_{\alpha_1\alpha_n} - \rho_0^{n-1}c^{(n)}_{\alpha_1\ldots\alpha_n}(\mathbf{k}_1,...,\mathbf{k}_{n-1})\right] \tag{S32}$$

We note that we use a summation convention for repeated indices. Then, the functional identity Eq. Eq. (S14) generalises to

$$\int d3 K^{(2)}_{\alpha\gamma}(1,3)H^{(2)}_{\gamma\beta}(3,2) = \delta_{\alpha\beta}(1,2) \tag{S33}$$

which in Fourier space after assuming translational and rotational invariance will read

$$K^{(2)}_{\alpha\gamma}(|\mathbf{k}_1|)H^{(2)}_{\gamma\beta}(|\mathbf{k}_1|) = \delta_{\alpha\beta} \tag{S34}$$

which is precisely a matrix inverse equation and thus we may write: $K^{(2)}_{\alpha\beta} = (H^{(2)})^{-1}_{\alpha\beta}$.

B.2. Multicomponent three-body correlation functions : $c^{(3)}_{\alpha\beta\gamma}$ and $S^{(3)}_{\alpha\beta\gamma}$. We need to consider the functional derivative of Eq. Eq. (S33) with respect to $\rho^{(1)}_\epsilon(4)$. We find

$$\begin{aligned} 0 &= \frac{\delta}{\delta\rho^{(1)}_\epsilon(4)}\int d3 K^{(2)}_{\alpha\gamma}(1,3)H^{(2)}_{\gamma\beta}(3,2) \\ &= \int d3 K^{(3)}_{\alpha\gamma\epsilon}(1,3,4)H^{(2)}_{\gamma\beta}(3,2) + \int d3d5 K^{(2)}_{\alpha\gamma}(1,3)H^{(3)}_{\gamma\beta\lambda}(3,2,5)K^{(2)}_{\lambda\epsilon}(5,4) \end{aligned} \tag{S35}$$

which in Fourier space will read

$$0 = K^{(3)}_{\alpha\gamma\epsilon}(\mathbf{k}_2,\mathbf{k}_3)H^{(2)}_{\gamma\beta}(\mathbf{k}_2) + K^{(2)}_{\alpha\gamma}(\mathbf{k}_1)H^{(3)}_{\gamma\beta\lambda}(\mathbf{k}_1,\mathbf{k}_2)K^{(2)}_{\lambda\epsilon}(\mathbf{k}_1+\mathbf{k}_2). \tag{S36}$$

To rearrange for $H^{(3)}$ we make use of the matrix inverse relation determined above. We also use that $K^{(2)}$ is a left and right inverse matrix to $H^{(2)}$, which holds true since all our tensors are symmetric in their indices (provided that we also accordingly exchange the corresponding Fourier modes). We then find that

$$H^{(3)}_{\alpha\beta\gamma}(\mathbf{k}_1,\mathbf{k}_2) = -H^{(2)}_{\alpha\mu}(\mathbf{k}_1)H^{(2)}_{\beta\nu}(\mathbf{k}_2)H^{(2)}_{\gamma\eta}(\mathbf{k}_1+\mathbf{k}_2)K^{(3)}_{\mu\nu\eta}(\mathbf{k}_1,\mathbf{k}_2) \tag{S37}$$

From which we derive after switching indices to that of the main text

$$S^{(3)}_{\alpha\beta\gamma}(\mathbf{k}_1,\mathbf{k}_2) = S^{(2)}_{\alpha\alpha'}(|\mathbf{k}_1|)S^{(2)}_{\beta\beta'}(|\mathbf{k}_2|)S^{(2)}_{\gamma\gamma'}(|\mathbf{k}_1+\mathbf{k}_2|)\left[\frac{\delta_{\alpha'\beta'}\delta_{\alpha'\gamma'}}{x^2_{\alpha'}} + \rho_0^2 c^{(3)}_{\alpha'\beta'\gamma'}(\mathbf{k}_1,\mathbf{k}_2)\right] \tag{S38}$$

where we have defined $x_\alpha = N_\alpha/N$ the concentration of species α. This result is equivalent to the result from (1) up to a minor different in the sense that their expression contains a redundant Kronecker-δ upon expanding the expression.

B.3. Multicomponent four-body correlation functions : $c^{(4)}_{\alpha\beta\gamma\sigma}$ and $S^{(4)}_{\alpha\beta\gamma\sigma}$. Repeating the same procedure, we consider the functional derivative of Eq. Eq. (S35) w.r.t $\rho^{(1)}_{\eta}(6)$. We then find that

$$
\begin{aligned}
0 = &\int d3 K^{(4)}_{\alpha\sigma\epsilon\eta}(1,3,4,6) H^{(2)}_{\gamma\beta}(3,2) \\
&+ \int d3d5 K^{(3)}_{\alpha\gamma\epsilon}(1,3,4) H^{(3)}_{\gamma\beta\lambda}(3,2,5) K^{(2)}_{\lambda\eta}(5,6) \\
&+ \int d3d5 K^{(3)}_{\alpha\gamma\eta}(1,3,6) H^{(3)}_{\gamma\beta\lambda}(3,2,5) K^{(2)}_{\lambda\epsilon}(5,4) \\
&+ \int d3d5d7 K^{(2)}_{\alpha\gamma}(1,3) H^{(4)}_{\gamma\beta\lambda\mu}(3,2,5,7) K^{(2)}_{\mu\eta}(7,6) K^{(2)}_{\lambda\epsilon}(5,4) \\
&+ \int d3d5 K^{(2)}_{\alpha\gamma}(1,3) H^{(3)}_{\gamma\beta\lambda}(3,2,5) K^{(3)}_{\lambda\epsilon\eta}(5,4,6).
\end{aligned} \tag{S39}
$$

which in Fourier space will lead to the following algebraic expression: l

$$
\begin{aligned}
0 = &K^{(4)}_{\alpha\sigma\epsilon\eta}(\mathbf{k}_1,\mathbf{k}_2,\mathbf{k}_3) H^{(2)}_{\gamma\beta}(\mathbf{k}_1+\mathbf{k}_2+\mathbf{k}_3) \\
&+ K^{(3)}_{\alpha\gamma\epsilon}(\mathbf{k}_1,\mathbf{k}_2) H^{(3)}_{\gamma\beta\lambda}(\mathbf{k}_1+\mathbf{k}_2,\mathbf{k}_3) K^{(2)}_{\lambda\eta}(\mathbf{k}_3) \\
&+ K^{(3)}_{\alpha\gamma\eta}(\mathbf{k}_1,\mathbf{k}_3) H^{(3)}_{\gamma\beta\lambda}(\mathbf{k}_1+\mathbf{k}_3,\mathbf{k}_2) K^{(2)}_{\lambda\epsilon}(\mathbf{k}_2) \\
&+ K^{(2)}_{\alpha\gamma}(\mathbf{k}_1) H^{(4)}_{\gamma\beta\lambda\mu}(\mathbf{k}_1,\mathbf{k}_2,\mathbf{k}_3) K^{(2)}_{\mu\eta}(\mathbf{k}_3) K^{(2)}_{\lambda\epsilon}(\mathbf{k}_2) \\
&+ K^{(2)}_{\alpha\gamma}(\mathbf{k}_1) H^{(3)}_{\gamma\beta\lambda}(\mathbf{k}_1,\mathbf{k}_2+\mathbf{k}_3) K^{(3)}_{\lambda\epsilon\eta}(\mathbf{k}_2,\mathbf{k}_3)
\end{aligned} \tag{S40}
$$

To rearrange in favour of $H^{(4)}$ we must multiply through by $H^{(2)}_{\epsilon\nu}(\mathbf{k}_2) H^{(2)}_{\eta\sigma}(\mathbf{k}_3)$ from the right and by $H^{(2)}_{\rho\alpha}(\mathbf{k}_1)$ from the left, which eventually gives after expressing quantities in terms of many-body structure factors and many-particle direct correlation functions and switching indices:

$$
\begin{aligned}
S^{(4)}_{\alpha\beta\gamma\sigma}(\mathbf{k}_1,\mathbf{k}_2,\mathbf{k}_3) = &S^{(2)}_{\alpha\alpha'}(k_1)\left(\frac{\delta_{\alpha'\beta'}\delta_{\alpha'\gamma'}}{x^2_{\alpha'}} + \rho_0^2 c^{(3)}_{\alpha'\beta'\gamma'}(\mathbf{k}_1,\mathbf{k}_2)\right) S^{(3)}_{\beta'\beta\sigma}(\mathbf{k}_1+\mathbf{k}_2,\mathbf{k}_3) S^{(2)}_{\gamma'\gamma}(k_2) \\
&+ S^{(2)}_{\alpha\alpha'}(k_1)\left(\frac{\delta_{\alpha'\beta'}\delta_{\alpha'\sigma'}}{x^2_{\alpha'}} + \rho_0^2 c^{(3)}_{\alpha'\beta'\sigma'}(\mathbf{k}_1,\mathbf{k}_3)\right) S^{(3)}_{\beta'\beta\gamma}(\mathbf{k}_1+\mathbf{k}_3,\mathbf{k}_2) S^{(2)}_{\sigma'\sigma}(k_3) \\
&+ S^{(3)}_{\alpha\beta\beta'}(\mathbf{k}_1,\mathbf{k}_2+\mathbf{k}_3)\left(\frac{\delta_{\beta'\gamma'}\delta_{\beta'\sigma'}}{x^2_{\beta'}} + \rho_0^2 c^{(3)}_{\beta'\gamma'\sigma'}(\mathbf{k}_2,\mathbf{k}_3)\right) S^{(2)}_{\gamma'\gamma}(k_2) S^{(2)}_{\sigma'\sigma}(k_3) \\
&- S^{(2)}_{\alpha\alpha'}(k_1)\left(\frac{2\delta_{\alpha'\beta'}\delta_{\alpha'\gamma'}\delta_{\alpha'\sigma'}}{x^3_{\alpha'}} - \rho_0^3 c^{(4)}_{\alpha'\beta'\gamma'\sigma'}(\mathbf{k}_1,\mathbf{k}_2,\mathbf{k}_3)\right) S^{(2)}_{\beta'\beta}(|\mathbf{k}_1+\mathbf{k}_2+\mathbf{k}_3|) S^{(2)}_{\gamma\gamma'}(k_2) S^{(2)}_{\sigma\sigma'}(k_3)
\end{aligned}
$$

where by neglecting all direct correlation functions we find that

$$
\begin{aligned}
S^{(4)}_{\alpha\beta\gamma\sigma}(\mathbf{k}_1,\mathbf{k}_2,\mathbf{k}_3) \approx &\frac{1}{x^2_{\alpha}} S^{(2)}_{\alpha\alpha'}(k_1) S^{(3)}_{\alpha'\beta\sigma}(\mathbf{k}_1+\mathbf{k}_2,\mathbf{k}_3) S^{(2)}_{\alpha'\gamma}(k_2) \\
&+ \frac{1}{x^2_{\alpha}} S^{(2)}_{\alpha\alpha'}(k_1) S^{(3)}_{\alpha'\beta\gamma}(\mathbf{k}_1+\mathbf{k}_3,\mathbf{k}_2) S^{(2)}_{\alpha'\sigma}(k_3) \\
&+ \frac{1}{x^2_{\beta'}} S^{(3)}_{\alpha\beta\beta'}(\mathbf{k}_1,\mathbf{k}_2+\mathbf{k}_3) S^{(2)}_{\beta'\gamma}(k_2) S^{(2)}_{\beta'\sigma}(k_3) \\
&- \frac{2}{x^3_{\alpha'}} S^{(2)}_{\alpha'\alpha}(k_1) S^{(2)}_{\alpha'\alpha}(|\mathbf{k}_1+\mathbf{k}_2+\mathbf{k}_3|) S^{(2)}_{\alpha'\gamma}(k_2) S^{(2)}_{\alpha'\sigma}(k_3)
\end{aligned} \tag{S41}
$$

The higher-order convolution approximations for multi-component systems may be derived similarly.

B. Additional Numerical details

As alluded to in the main text, there is a fundamental limit on the resolution with which we can choose the $\mathbf{k}$-vectors at which we want to probe the density modes. Specifically, the set of allowed $\mathbf{k}$-vectors is constrained to $2\pi/L\,[n_x, n_y, n_z]^T$, with n_x, n_y, and n_z integers. Since both time and space are now discretised, we can introduce the complex numbers $\rho_{n,i} = \rho(\mathbf{k}_n, t_i)$ where n is an index that uniquely combines the spatial indices n_x, n_y, n_z. All many-body structure factors can now be expressed in terms of this quantity as simple contractions.

For example, the time-average two- and three-point static structure factor become

$$S^{(2)}(\mathbf{k}_n) = \frac{1}{NN_t}\sum_{i=1}^{N_t} \rho_{n,i}\rho^*_{n,i} \qquad \text{[S42]}$$

$$S^{(3)}(\mathbf{k}_n, \mathbf{k}_m) = \frac{1}{NN_t}\sum_{i=1}^{N_t} \rho_{n,i}\rho_{m,i}\rho^*_{n+m,i}, \qquad \text{[S43]}$$

where * denotes the complex conjugate, and N_t is the number of snapshots at which the density modes are sampled.

Assuming that the dispersion of particles is isotropic and translationally invariant, the resulting set of numbers can be binned to produce a quantity that is only dependent on $n-1$ wave numbers and $0, 1, 3, 6, 9, \ldots$ angles for $n = 2, 3, 4, 5, 6, \ldots$, respectively. This binning greatly increases the statistical power, since for high n, there typically exist many combinations of wave vectors $\mathbf{k}_i$ which end up in the same bin. Throughout this work, we choose the one-sided bin widths to be $\Delta kD = 0.1$, $\Delta\cos\theta = 0.05$ and $\Delta\phi = 0.05\pi$ for the wave number, polar and azimuthal angle respectively. For the case of Fig. 1a from the main text, this results in roughly 10^5 different sets of wave vectors to be averaged over for each point on the sphere. We use the same binning procedure for the convolution approximation, to make sure any differences we find are not caused by the discretisation. We exclude any cases where a subset of the n wave vectors sum to the zeros vector. A special case of this is the diagonal four-point correlation function, which we analyse separately.

For correlation functions of three or more density modes, evaluating the numbers $\rho_{n,i}$ first and subsequently contracting them to find the correlation functions significantly reduces the total computational cost compared to a more direct evaluation method. In particular, the full calculation needed to produce Fig. 1 of the main text from a set of trajectories of $N = 10^3$ particles saved at $N_t = 10^3$ time points takes roughly thirty minutes to complete on a modern laptop cpu.

C. Additional Numerical Results

We present below additional results for the three- and four-body structural correlation functions in both the liquid and the supercooled regime.

A. Three-Body Correlations. The triplet-correlation function forms the first step in identifying non-trivial preferred local structural configurations in liquids. Previous studies (3, 4) have shown that preferred angles exist in various Kob-Andersen mixtures, and that they can be related to maxima in the many-body structure factors. Specifically, earlier work by Coslovich focused primarily on the angular dependence of the equal-wavelength triplet structure factor, *i.e.*, $S^{(3)}(k_1 = k, k_2 = k, \cos\theta_{12})$. (3) Indeed, these equal wavelength triplet structure factors show a non-trivial angular dependence that can be qualitatively related to the preferred local structure in real space. We present results that show complete qualitative agreement with those of Coslovich, even though we consider a different model system. However, we have also evaluated the triplet correlations for unequal wave numbers, of which a subset is shown in the main text, and observe that the preferred angles depend drastically on the combination of wave vectors that is probed, as is expected on the grounds of real-space intuition.

As Coslovich found, the convolution approximation agrees very well with the simulation results, only qualitatively failing if the angle between the wave-vectors is very small, in which case the three-particle direct correlations apparently become important. This failure of the convolution approximation exists both in the low-density liquid regime and in the high-density supercooled regime, although it is more pronounced in the latter.

We have scanned the full phase space, which we show in Fig. S1, but observe no clear qualitative changes in the triplet structure as the density is increased from that of a regular liquid to that of a deeply supercooled one. As we show in the main text, this is in direct opposition to the case of quartet-structures, encoded in $S^{(4)}$, where there are clear qualitative changes as the glass transition is approached.

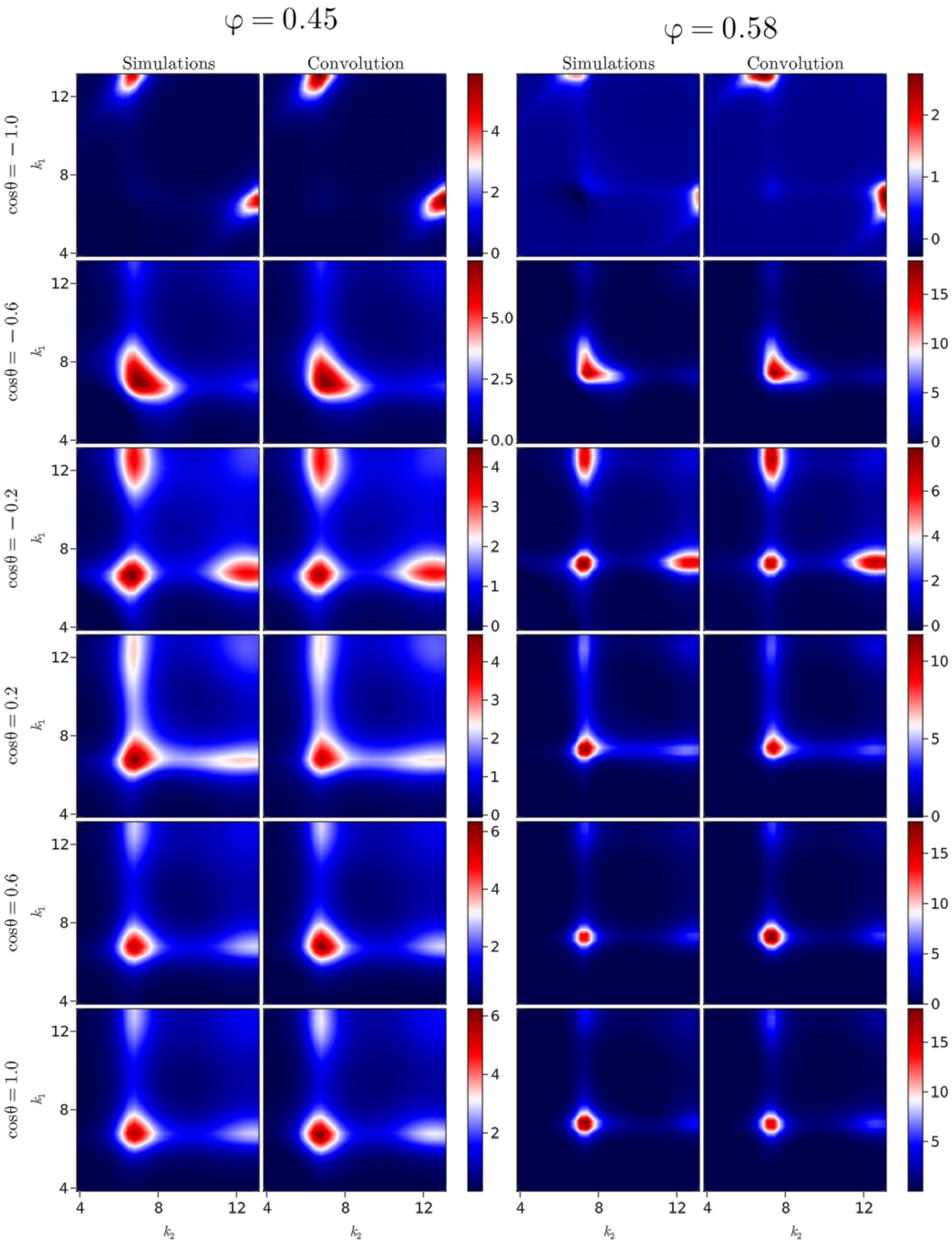

Fig. S1. Triplet correlation function $S^{(3)}(k_1, k_2, \theta_{12})$ as a function of k_1 and k_2 for different values of θ_{12} in different rows. The left two columns compare simulation results and the convolution approximation for low-density liquids, whereas the right compare them for supercooled systems. The colours are such that the corresponding simulation results and convolution approximation share the same colour scheme.

B. Four-Body Correlations. In this section we present additional results on the four-body structure factor, for $k_3D = 9.5$ and $k_3D = 4.0$, not shown in the main article. These provide additional evidence for the conclusions in the main text that the convolution approximation agrees with simulations excellently at high k (see Fig. S2 for the instance of $k_3D = 9.5$), but progressively starts to fail qualitatively once the wave length is increased. The failure of the convolution approximation is already seen at $k_3D = 4.0$, see Fig. S3(b,d,f), but is even more prominent in for $k_3D = 2.0$, see Fig. 1 of the main text. For all our analyses we have fixed $k_1D = k_2D = 7.2$ in order to retain a good signal-to-noise ratio.

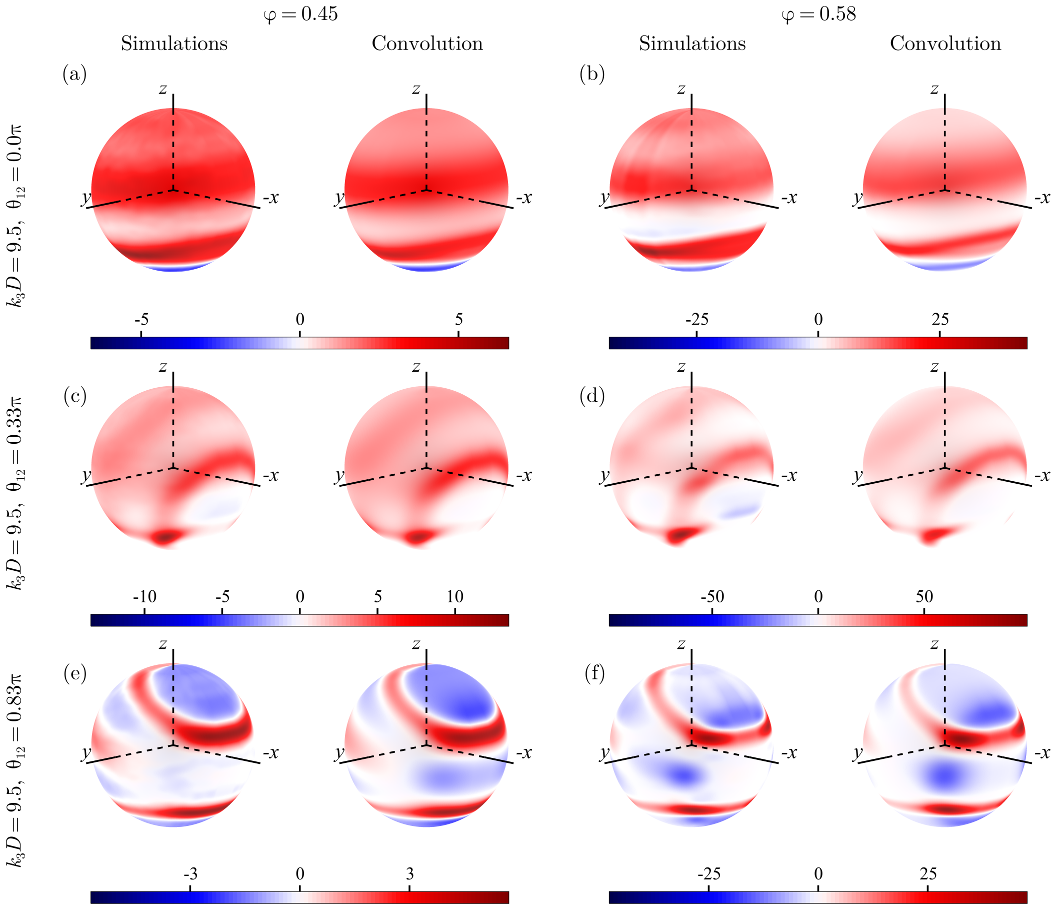

Fig. S2. Quartet correlation function $S^{(4)}(k_1, k_2, k_3, \theta_{12}, \theta_{13}, \phi_{23})$ as a function of θ_{13} and ϕ_{23} for different values of θ_{12} in different rows, given that $k_1D = k_2D = 7.2$, and $k_3D = 9.5$, the latter coinciding with the first minimum of the standard structure factor. The left two columns compare simulation results and the convolution approximation for low-density liquids, whereas the right compare them for supercooled systems. The colours are such that the corresponding simulation results and convolution approximation share the same colour scheme.

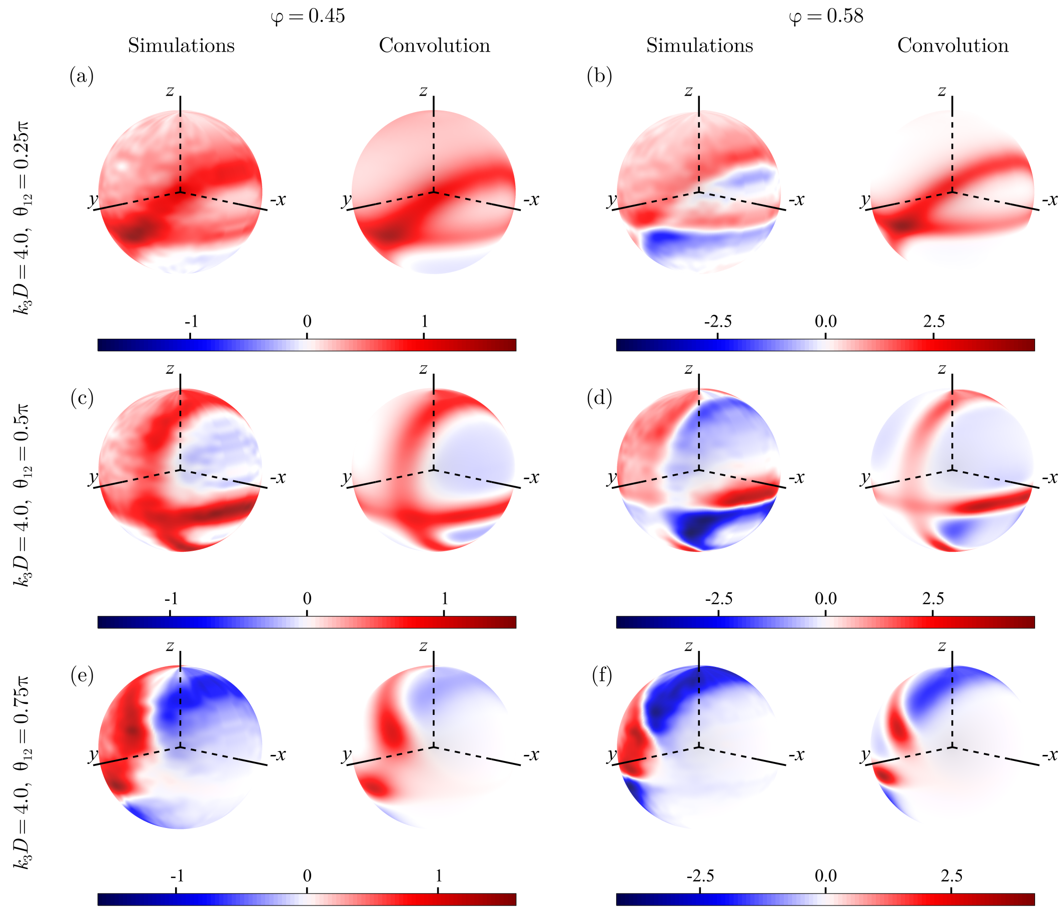

Fig. S3. Quartet correlation function $S^{(4)}(k_1, k_2, k_3, \theta_{12}, \theta_{13}, \phi_{23})$ as a function of θ_{13} and ϕ_{23} for different values of θ_{12} in different rows, given that $k_1D = k_2D = 7.2$, and $k_3D = 4.0$. The left two columns compare simulation results and the convolution approximation for low-density liquids, whereas the right compare them for supercooled systems. The colours are such that the corresponding simulation results and convolution approximation share the same colour scheme.

References

1. J Barrat, J Hansen, G Pastore, On the equilibrium structure of dense fluids: triplet correlations, integral equations and freezing. *Mol. Phys.* **63**, 747–767 (1988).
2. G Biroli, JP Bouchaud, K Miyazaki, DR Reichman, Inhomogeneous mode-coupling theory and growing dynamic length in supercooled liquids. *Phys. Rev. Lett.* **97** (2006).
3. D Coslovich, Static triplet correlations in glass-forming liquids: A molecular dynamics study. *The J. chemical physics* **138**, 12A539 (2013).
4. V Levashov, R Ryltsev, N Chtchelkatchev, Structure of the simple harmonic-repulsive system in liquid and glassy states studied by the triple correlation function. *J. Physics: Condens. Matter* **33**, 025403 (2020).
